# Association of losartan with outcomes in metastatic pancreatic cancer patients treated with chemotherapy

**Published:** 2021-03-24

**Authors:** Anup Kasi, Jessica Allen, Kathan Mehta, Prasad Dandawate, Subhrajit Saha, Stefan Bossmann, Shrikant Anant, Weijing Sun

**Affiliations:** ^1^University of Kansas Medical Center Division of Medical Oncology, Kansas, USA; ^2^University of Kansas School of Medicine, Kansas, USA; ^3^University of Kansas School of Medicine Division of Cancer Biology, Kansas, USA; ^4^University of Kansas School of Medicine Division of Radiation Oncology, Kansas, USA

**Keywords:** angiotensin II receptor antagonists, angiotensin receptor blockers, losartan, metastatic pancreatic cancer, pancreatic cancer

## Abstract

**Background:**

Previous trials have shown improved efficacy of neoadjuvant treatment when combined with angiotensin II receptor antagonist, losartan in patients with locally advanced pancreatic ductal adenocarcinoma (PDA). However, role of losartan is unknown in metastatic PDA. In this retrospective observational study, we examined the relationship between losartan use at time of diagnosis and continued through chemotherapy treatment with clinical outcomes in patients with metastatic PDA that received chemotherapy.

**Methods:**

We retrospectively evaluated 114 metastatic PDA patients treated at University of Kansas Cancer Center between January 2000 and November 2019. We compared overall survival (OS), progression-free survival (PFS), objective response rate (ORR), and disease control rate (DCR) between patients using losartan at time of their cancer diagnosis and a control group of patients who were not on losartan. A subgroup analysis was performed based on patients who were on a 100 mg dose of losartan along with chemotherapy versus patients treated with chemotherapy (without losartan). Another subgroup analysis was performed based on chemotherapy regimen: Fluorouracil, leucovorin, oxaliplatin, and irinotecan (FOLFIRINOX) versus Gemcitabine and Abraxane.

**Results:**

No significant difference was found in OS (p=0.466) or PFS (p=0.919) in patients on losartan (median 274 day, 83 day) and control patients (median 279 day, 111 day). No significant difference was found in ORR (p=0.621) or in DCR (p=0.497). No significant difference was found in OS (p=0.771) or PFS (p=0.0604) in losartan patients (median 347 day, 350 day) and control patients (median 333 day, 101 day) treated with FOLFIRINOX. No significant difference was found in OS (p=0.916) or PFS (p=0.341) in losartan (median 312 day, 69 day) and control patients (median 221 day, 136 day) treated with Gemcitabine plus Abraxane. No significant difference was found in OS (p=0.727) or PFS (p=0.790) in 100 mg losartan patients (median 261 day, 84 day) and control (median 279 day, 111 day).

**Conclusions:**

Patients on losartan at time of diagnosis and continued through chemotherapy treatment had no significant difference in OS, PFS, ORR, DCR than control patients. Subgroup analysis of patients treated with FOLFIRINOX revealed a longer PFS with losartan than control but did not reach statistical significance, likely due to small sample size. Our findings should be validated in a larger cohort to confirm if the benefit of losartan and FOLFIRINOX seen in a neoadjuvant setting for locally advanced cancer also applies to metastatic cancer.

**Relevance for Patients:**

This research adds to growing data on the efficacy of angiotensin receptor blocking drugs as adjunctive treatment in addition to chemotherapy in pancreatic cancer with specific focus on metastatic disease.

## 1. Introduction

Pancreatic cancer is the eleventh most common form of cancer in the United States, representing 3.2% of new cancer diagnoses [1]. It is currently the third leading cause of cancer-related death in the US, with a 5-year survival rate of 9% across all stages [1,2]. The 5-year survival drops to 3% for patients diagnosed after the disease has reached metastatic stage, which unfortunately is the case for over half of patients with new pancreatic cancer diagnoses [2]. Patients diagnosed with disease in the localized or regional stage fare better, with 5-year survival rates of 39.4% and 13.3%, respectively, due to the increased likelihood of safe and successful resection of tumors at these stages [1]. The 5-year survival rate of pancreatic cancer has increased from 6% to 10% over the last decade, and while this is progress, the 5-year survival remains low in comparison to other common malignancies despite advances in cancer therapy in the last few decades [3,4].

One explanation for the consistently low survival rate of pancreatic cancer is a lack of screening methods for detection of the disease in early stages [2]. It is referred to as a “silent disease,” because pancreatic cancer rarely presents with specific signs until reaching advanced stages. Common prodromal symptoms include jaundice, upper abdominal or back pain, pancreatitis, stomach bloat, limb swelling secondary to blood clots, weakness, and nausea, all of which typically occur once the tumor is large enough to either disrupt pancreatic function, compress nearby structures, or spread to distant organs [2]. Thus, there is need for efficient methods of early detection, prognostic markers to guide treatment decisions, and treatments with greater efficacy in increasing overall survival (OS).

In an effort to address this need, a previous study showed improved efficacy of neoadjuvant treatment with fluorouracil, leucovorin, oxaliplatin, and irinotecan (FOLFIRINOX) when combined with the angiotensin II receptor antagonist (ARB), losartan, followed by chemoradiotherapy at time of diagnosis in patients with locally advanced pancreatic ductal adenocarcinoma (PDA) [5]. Murphy *et al*. demonstrated a margin-negative resection rate statistically higher than expected, highlighting a promising insight in treatment of locally advanced pancreatic cancer. In addition, a separate study found an independent association between patients with non-metastatic PDA and chronic angiotensin system inhibitor (ACEi) use and longer OS [6]. The efficacy of losartan in pancreatic cancer is most likely explained by its role in inhibiting signaling pathways associated with development of fibrotic tumor microenvironments [7]. Fibrotic tumor microenvironments are associated with poorer treatment outcomes [7]. Angiotensin II receptor I blockers, such as losartan, inhibit the transforming growth factor (TGF-b) pathway, a known activator of fibroblasts associated with the development of fibrosis in cancer [7].

The effect of renin-angiotensin system (RAS)-modulating drugs on treatment outcomes in pancreatic cancer can be explained by the role RAS plays in the tumor microenvironment [8]. RAS is best known as an enzymatic system responsible for the regulation of blood volume and systemic vascular resistance. However, many tissues express a localized form of RAS with primary effect at the cellular level [7]. Multiple preclinical studies have implicated RAS signaling in tumor growth through alteration of tumor desmoplasia, vasculature, inflammation, and immune cells [8]. Specifically, in regard to tumor angiogenesis, progressively more evidence suggests that angiotensin II/angiotensin II type I receptor (AT1R) signaling enhances VEGF-mediated angiogenesis, which, in turn, promotes tumor hypoxia and compensatory dissemination [8]. Thus, RAS-modulating drugs represent a promising avenue in the treatment of metastatic PDA, because inhibiting the enhancement of angiogenesis by RAS could dampen the cellular drive for metastasis.

Given the implications of RAS in tumor advancement shown by preclinical studies, in addition to the enhanced efficacy of neoadjuvant treatment in locally advanced disease when combined with losartan, it is possible that use of losartan or other RAS-modulating drug may alter the course of tumor growth and spread in pancreatic cancer patients diagnosed at the metastatic stage. In this observational retrospective study, we analyzed the effect of losartan use at time of diagnosis on OS, progression-free survival (PFS), objective response rate (ORR), and disease control rate (DCR) in metastatic pancreatic cancer patients treated with chemotherapy at the University of Kansas Cancer Center (KUCC) to examine the implication of RAS-modulating drugs on metastatic disease.

## 2. Materials and Methods

This study was an observational retrospective chart review of the characteristics of 114 patients with metastatic PDA diagnosed between January 2000 and November 2019 and treated with chemotherapy from the University of Kansas Cancer Center medical records. Demographic data, such as age, gender, race, smoking status, ECOG status, tumor location, and treatment received were also collected. Patient groups based on losartan use at time of metastatic pancreatic cancer diagnosis were identified through KUCC’s medical informatics system, HERON [9,10]. Chart review was then conducted to ensure losartan was used at time of diagnosis and continued throughout treatment. Patient data were stored on a secure REDCAPs database. All data were deidentified before analysis.

Primary outcomes of interest included OS, PFS, ORR, and DCR. The outcomes between patients using ARB losartan and a control group of patients not on losartan were compared using log-rank trends tests and Kaplan–Meier survival curves. A subgroup analysis assessing OS and PFS was conducted between patients on high-dose losartan, defined as 100 mg daily based on the maximum dose for hypertension in adults, at diagnosis versus control patients [11]. Another subgroup analysis of OS and PFS was conducted between patients on losartan that was primarily treated with the chemotherapy regimen FOLFIRINOX versus control patients not on losartan treated with FOLFIRINOX and patients on losartan treated with gemcitabine and Abraxane versus control patients treated with gemcitabine and Abraxane.

## 3. Results

### 3.1. Characteristics of patients

A total of 114 patients with metastatic pancreatic cancer diagnosed between January 2000 and November 2019 from the University of Kansas Cancer Center and treated with chemotherapy were included in the study. Patients were divided into groups based on use of losartan at time of PDA diagnosis, with sub-groups based on dose of losartan at time of diagnosis. Demographic characteristics within each group are listed in Table 1.

**Table 1:** Baseline demographic characteristics between experimental groups

Characteristics	25 mg Losartan	50 mg Losartan	100 mg Losartan	Unknown dose Losartan	Control
Number	7	20	28	2	57
Age (median)	68	69	67	68	61
Gender (%)					
Male	42.9%	65.00%	50.00%	50.00%	56.1%
Female	57.10%	35.00%	50.00%	50.00%	43.9%
Race					
White	5 (71.4%)	12 (60.0%)	19 (67.9%)	2 (100.0%)	47 (82.5%)
Black or African American	2 (28.6)	5 (25.0%)	4 (14.3%)	0 (0.0%)	7 (12.3%)
Other	0 (0.0%)	1 (5.0%)	4 (14.3%)	0 (0.0%)	3 (5.3%)
Smoking Status					
Yes	5 (71.4%)	12 (60.0%)	13 (46.4%)	2 (100.0%)	35 (61.4%)
No	2 (28.6)	8 (40.0%)	15 (53.6%)	0 (0.0%)	21 (36.8%)
ECOG Status					
0-1	3 (42.9%)	15 (75.0%)	18 (64.3%)	2 (100.0%)	45 (79.0%)
2 or higher	2 (28.6)	0 (0.0%)	4 (14.3%)	0 (0.0%)	12 (21.1%)
Tumor location					
Head	4 (57.1%)	15 (75.0%)	19 (67.9%)	0 (0.0%)	31 (54.4%)
Body	2 (28.6%)	0 (0.0%)	4 (14.3%)	1 (50.0%)	12 (21.1%)
Tail	0 (0.0%)	3 (15.0%)	4 (14.3%)	1 (50.0%)	14 (24.6%)
Neck	1 (14.3%)	1 (5.0%)	0 (0.0%)	0 (0.0%)	0 (0.0%)
Baseline CA19-9					
Normal (<38)	1 (14.3%)	2 (10.0%)	4 (14.3%)	0 (0.0%)	9 (15.8%)
Abnormal	5 (71.4%)	18 (90.0%)	20 (71.4%)	2 (100.0%)	44 (77.2%)
Treatment received					
FOLFIRINOX	0 (0.0%)	4 (20.0%)	9 (32.1%)	1 (50.0%)	28 (49.1%)
Gemcitabine plus albumin-bound Paclitaxel (Abraxane)	5 (71.4%)	7 (35.0%)	11 (39.3%)	1 (50.0%)	23 (40.4%)
Other	2 (28.6%)	9 (45.0%)	8 (28.6%)	0 (0.0%)	6 (10.5%)

FREQ procedure was done to test for association between covariates such as gender and losartan use (p=0.851), race and losartan use (p=0.323), and smoking and losartan use (p=0.492). No significant association was found between any of these variables and losartan use, indicating even distribution between groups. Conversely, FREQ procedure performed to test association between chemotherapy regimens (FOLFIRINOX, gemcitabine + Abraxane, gemcitabine, capecitabine, other regimen, and no regimen) and losartan use found significant differences in groups (p=0.0137). This explains the numerical difference between patients on losartan treated with FOLFIRINOX (14, or 24.6% of the total losartan sample) and control patients treated with FOLFIRINOX (28, or 49.1% of the total control sample).

TTEST procedure found a significant difference in age of the losartan group and the control group (p=0.0034). Control group patients had a lower median age (61 years) when compared to patients on losartan (68 years).

### 3.2. Efficacy

**Figure 1:**
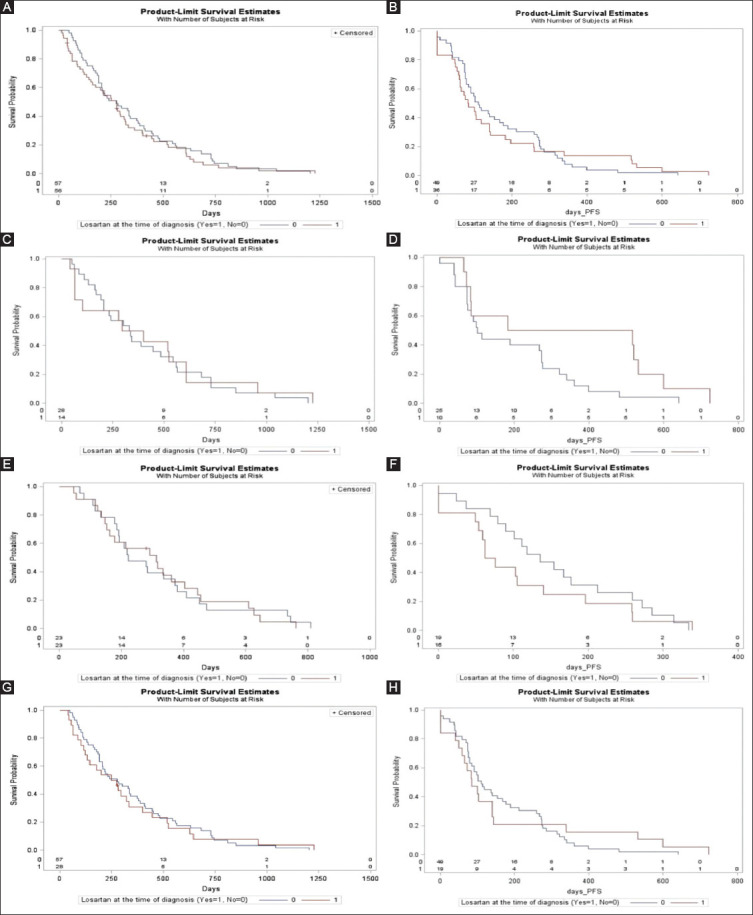
**(A)** Survival curve comparison of overall survival (OS) of losartan versus control groups. **(B)** Survival curve comparison of progression-free survival (PFS) of losartan versus control groups; **(C)** survival curve comparison of OS of FOLFIRINOX losartan versus FOLFIRINOX control groups; **(D)** survival curve comparison of PFS of FOLFIRINOX losartan versus FOLFIRINOX control groups; **(E)** survival curve comparison of OS of gemcitabine+abraxane losartan versus gemcitabine + Abraxane control groups; **(F)** survival curve comparison of OS of gemcitabine + Abraxane losartan versus gemcitabine + Abraxane control groups; **(G)** survival curve comparison of OS of 100 mg losartan versus control groups; **(H)** Survival curve comparison of PFS of 100 mg losartan versus control groups.

As shown in Table 2, the median OS for patients using losartan was 274 days and the median PFS for this group was 83 days. The median OS for control group patients not on losartan was 279 days while the median PFS was 111 days. No significant difference was found between the losartan group and the control group in OS (p=0.466) or PFS (p=0.919), as shown in Figure 1A and B.

**Table 2:** Log-rank trend tests of median OS, median PFS, DCR, and ORR in patients on losartan and patients not on losartan with additional subgroup analyses based on chemotherapy regimen

Group	Median OS (days)	p	Median PFS (days)	p	DCR p	ORR p
Losartan	274	0.466	83	0.919	0.497	0.621
Control	279		111			
Losartan+Gemcitabine+Abraxane	312	0.916	69	0.314		
Gemcitabine+Abraxane without Losartan	221		136			
Losartan+FOLFIRINOX	347	0.916	350	0.0604		
FOLFIRINOX without Losartan	333		101			
Losartan 100 mg	261	0.727	84	0.790		
Control	279		11			

No significant difference was found in ORR (p=0.621) or in DCR (p=0.497) between losartan and control groups, as shown in Table 2

In a subgroup analysis of patients on losartan treated with the chemotherapy regimen FOLFIRINOX and control patients treated with FOLFIRINOX, the median OS for the losartan group was 347 days and median PFS was 350 days, as shown in Table 2. Median OS in the control group was 333 days and median PFS was 101 days, as shown in Table 2. No significant difference was found in OS (p=0.916) or PFS (p=0.0604), as shown in Figure 1C and D.

Subgroup analysis of patients on losartan treated with the chemotherapy regimen of gemcitabine plus Abraxane and control patients treated with gemcitabine plus Abraxane was also performed. In the losartan group, median OS was 312 days and median PFS was 69 days, as shown in Table 2. In the control group, median OS was 221 days and median PFS was 136 days, as shown in Table 2. No significant difference was found between groups in OS (p=0.916) and PFS (p=0.341), as shown in Figure 1E and F.

In another subgroup analysis between patients on high-dose losartan and control patients, the median OS for high-dose losartan patients was 261 days and the median PFS was 84 days, as shown in Table 2. The median OS for control patients was 279 days and the median PFS was 111 days, as shown in Table 2. No significant difference was found in OS (p=0.727) or PFS (p=0.790), as shown in Figure 1G and H.

## 4. Discussion

In this study, we found no statistically significant difference in OS and PFS between patients with metastatic pancreatic cancer using losartan and control group patients not on losartan. There was no significant difference in ORR and DCR between patients on losartan and control group patients. Both subgroup analyses based on chemotherapy regimen, FOLFIRINOX and gemcitabine plus Abraxane, found no statistically significant difference in OS and PFS between experimental groups and control groups. However, there was notable numerical difference in PFS in the FOLFIRINOX group, with a median PFS of 350 days in the losartan group compared with a median PFS of 101 days in the control group. There was no significant difference seen in OS and PFS in patients on the maximum dose of losartan and control patients.

These findings are overall in agreement with a study conducted by Hao *et al.*, which found no association between the use of ACEi and improved OS in patients with metastatic pancreatic cancer through retrospective analysis [6]. Our findings differ from those of Nakai *et al.*, which found that the use of an ACEi or ARB was associated with significantly increased OS and PFS in a mixed cohort of locally advanced and metastatic PDA patients in both a retrospective analysis (n=155) and a Phase I trial (n=14) [12,13]. This difference could be explained by the heterogeneity of the patient population analyzed by Nakai *et al.*, as their study included a mix of locally advanced and metastatic PDA [13]. In addition, Nakai *et al*. only studied patients treated with gemcitabine [12]. Given that our study exclusively involved metastatic patients, our findings increase evidence that the survival benefit of ARBs seen in locally advanced PDA may not translate to metastatic PDA.

### 4.1. Limitations

Limitations of this study include retrospective chart review design, which only allows identification of correlation between losartan use and increased PFS though it did not meet statistical significance. Another limitation was the small size of analysis (n=114), which reduces the study’s power. Other limitations include differences in demographic characteristics, such as age, between experimental and control groups, lack of collection of other demographic characteristics such as alcohol use, and possible homogeneity due to sample selection from only one treatment center which limits generalizability of results.

A specific limitation in the subgroup analysis between patients on losartan treated with FOLFIRINOX and control patients treated with FOLFIRINOX is the numerical difference between subjects in each group. About 24.6% of the losartan group was treated with FOLFIRINOX while 49.1% of the control group was treated with FOLFIRINOX. This numerical difference (13 losartan + FOLFIRINOX patients vs. 28 control + FOLFIRINOX patients) could have affected the significance in difference in OS and PFS between these two groups.

### 4.2. Future directions

Our finding of numerically increased PFS in the losartan group treated with FOLFIRINOX warrants further investigation within a larger cohort with numerically equivalent groups. Given Murphy *et al*.’s findings that losartan enhanced efficacy of neoadjuvant FOLFIRINOX in locally advanced PDA patients, our findings of increased PFS in metastatic patients treated with FOLFIRINOX and losartan could give insight into a unique relationship between losartan and FOLFIRINOX specifically, as the same relationship was not seen in the gemcitabine plus Abraxane treatment group [5]. On the same note, the role of fibrotic tumor microenvironment may be different at a metastatic site when compared to the primary site of cancer in the pancreas.

Further investigation should be done to examine the relationship between losartan use and throughout treatment and OS and PFS in patients treated with FOLFIRINOX. Our findings show that losartan is a numerical prognostic factor of PFS, and these findings should be validated in a larger cohort and in the form of a prospective study. In addition, the combination of losartan and FOLFIRINOX with immunotherapy is worth investigating in locally advanced and metastatic pancreatic cancer populations. One ongoing trial is investigating this combination in localized pancreatic cancer [14]. Given losartan’s inhibition of TGF-beta induced fibrogenesis, it is possible that an additive effect in treatment efficacy could be seen when combined with immunotherapy’s ability to prevent blockage of the anti-tumor immune response in locally advanced and metastatic tumors.
